# Clinic and patient variation in intermediate clinical outcomes for type 2 diabetes: a multilevel analysis

**DOI:** 10.1186/s12875-019-1045-1

**Published:** 2019-11-15

**Authors:** Yvonne Mei Fong Lim, Swee Hung Ang, Nazrila Hairizan Nasir, Fatanah Ismail, Siti Aminah Ismail, Sheamini Sivasampu

**Affiliations:** 1 Institute for Clinical Research, National Institutes of Health, Ministry of Health Malaysia, No.1, Jalan Setia Murni U13/52, Setia Alam, Selangor Malaysia; 2 Julius Center for Health Sciences and Primary Care, University Medical Center Utrecht, Utrecht, the Netherlands; 3 Department of Social and Preventive Medicine, Faculty of Medicine, University of Malaya, Kuala Lumpur, Malaysia; 4 Family Health Development Division, Public Health Department, Ministry of Health Malaysia, Level 4, Block E6, Complex E, 62590 Putrajaya, Malaysia

**Keywords:** Type 2 diabetes, Intermediate outcomes, Multilevel analysis, Variation

## Abstract

**Background:**

Variation at different levels of diabetes care has not yet been quantified for low- and middle-income countries. Understanding this variation and its magnitude is important to guide policy makers in designing effective interventions. This study aims to quantify the variation in the control of glycated haemoglobin (HbA1c), systolic blood pressure (SBP) and low-density lipoprotein cholesterol (LDL-C) for type 2 diabetes (T2D) patients at the clinic and patient level and determine patient and clinic factors associated with control of these outcomes in T2D.

**Methods:**

This is a cross-sectional study within the baseline data from the impact evaluation of the Enhanced Primary Health Care (EnPHC) intervention on 40 public clinics in Malaysia. Patients aged 30 and above, diagnosed with T2D, had a clinic visit for T2D between 01 Nov 2016 and 30 April 2017 and had at least one HbA1c, SBP and LDL-C measurement within 1 year from the date of visit were included for analysis. Multilevel linear regression adjusting for patient and clinic characteristics was used to quantify variation at the clinic and patient levels for each outcome.

**Results:**

Variation in intermediate clinical outcomes in T2D lies predominantly (93% and above) at the patient level. The strongest predictors for poor disease control in T2D were the proxy measures for disease severity including duration of diabetes, presence of microvascular complications, being on insulin therapy and number of antihypertensives. Among the three outcomes, HbA1c and LDL-C results provide greatest opportunity for improvement.

**Conclusion:**

Clinic variation in HbA1c, SBP and LDL-C accounts for a small percentage from total variation. Findings from this study suggest that standardised interventions need to be applied across all clinics, with a focus on customizing therapy based on individual patient characteristics.

## Background

There is an estimated 424.9 million people with diabetes globally and about 80% live in low- and middle-income countries (LMIC) [1]. Over the past decade, prevalence of diabetes increased most rapidly in LMICs. Epidemiological transition in LMICs is distinct from high income countries because communicable diseases coexist with the rising epidemic of non-communicable diseases. Malaysia has a high prevalence of diabetes, where 17.5% of the population is affected compared to global estimates of 8.8% [1, 2]. Various strategies to improve diabetes care such as medication adherence clinic, diabetes education, revision of the clinical practice guidelines and diabetes audits [3–6] have been implemented in Malaysia but control of intermediate clinical outcomes including glycated haemoglobin (HbA1c), systolic blood pressure (SBP) and low-density lipoprotein cholesterol (LDL-C) has been suboptimal. The National Diabetes Registry, which captured data on diabetic patients from 644 public health clinics in all states of Malaysia, reported mean HbA1c of 8.1% in 2012 [7]. Only 40.9% achieved recommended blood pressure target of ≤130/80 mmHg and 37.8% achieved LDL-C levels of ≤2.6 mmol/L in the same year [7].

Variation in diabetes care is mainly described based on the concept that access and quality of care is highly dependent on where patients live and seek care. Understanding how health care facilities vary in diabetes process and outcome measures does not only allow for performance benchmarking, but also provide potential opportunities for quality improvement and cost reduction. Although not all geographical variation is inappropriate, the aim of diabetes care should be to minimise variation and maximise evidence-based practice [8]. Studies have quantified variation in diabetes outcomes at patient, physician, clinic and health system levels and a majority of these were based on data from the United States of America and other high-income European nations [8–10]. Diabetes outcomes from these countries may not necessarily be applicable to patients in countries with low- and middle-income economies because of differences in maturity of health systems and infrastructure. To our knowledge, variation in diabetes care has not yet been quantified for low- and middle-income settings like Malaysia.

Previous studies have investigated the association of facility and patient factors on intermediate clinical outcomes in diabetes [11–13] but few have examined how these outcomes differ within and between facilities. This concept considers the phenomenon of clustering of health outcomes within geographical locations [14]. Understanding the variation at different levels of care and its magnitude could provide useful information to guide policy makers in designing effective interventions. From a practical perspective, tailored quality improvement measures can be applied only to clinics which are poor performers in the case where diabetes outcomes are highly clustered within clinics. Conversely, in settings with low clustering among clinics, a single standardised intervention across all clinics would be more useful in improving overall diabetes outcomes.

It is also known that all diabetes quality indicators focus primarily on reducing diabetes complications through control of intermediate clinical measures of diabetes, which are primarily serum glucose, blood pressure and LDL-C [8]. Therefore, the objective of this study was to quantify the variation in the control of HbA1c, SBP and LDL-C for type 2 diabetes (T2D) patients at the clinic and patient level. We also aimed to determine the patient and clinic determinants are associated with control of these intermediate clinical outcomes in T2D.

## Methods

### Study design

This cross-sectional analysis was based on baseline data from a larger study entitled “Evaluation of the Enhanced Primary Healthcare (EnPHC) interventions in public health clinics” (EnPHC-Eva). The EnPHC-Eva was a quasi-experimental controlled study which aimed to determine the effectiveness of a multifaceted intervention package called EnPHC on process of care and intermediate clinical outcomes of patients with T2D and hypertension in 40 public health clinics in Malaysia. At the time of writing, the EnPHC-Eva has just completed post-intervention data collection and analysis. A study protocol for the EnPHC-Eva study is currently under journal review. Ethical approval was granted by the Medical Research Ethics Committee, Ministry of Health Malaysia (NMRR-17-267-34768).

### Setting

Malaysia has a dual-sector healthcare system; consisting of a public and private sector. The private sector is mainly funded by out-of-pocket payments and private insurance [15]. Health services in the public sector are heavily subsidised by general taxation and patients pay a small fee of between US$0.30 and US$ 4.50 for outpatient services, depending on citizenship status [15]. Hence, the public health sector manages the bulk of chronic conditions in the country [16]. For diabetes, patients mainly sought treatment at public clinics (59.3%), followed by public hospitals (20.0%), private clinics (15.1%), private hospitals (3.6%) and a remaining small percentage purchased medications from pharmacies or seeked traditional and alternative medicine [2].

The EnPHC interventions focused on public clinics because diabetes was largely managed in this healthcare setting. The clinics involved in this study were located in two states in Malaysia; Selangor and Johor. These two states were selected based on balance between regional representativeness, budget and implementation capacity. Each public health clinic was responsible for the care of the population residing within its assigned catchment area. Patients with diabetes were mainly managed by medical officers, who were licensed medical doctors with basic medical training. Some of them practice under the guidance of a family medicine specialist (FMS) who has formal postgraduate training in primary care practice, depending on whether there is a full-time or visiting FMS at their respective clinics. Specialised diabetes education and/or medication adherence clinic was available in some clinics. A diabetes educator provides individual or group-based education for diabetes patients on related topics which include healthy diet, foot care, exercise, self-monitoring, medication usage and goal setting and this role is usually performed by a nurse who has undergone formal training modules in diabetes care. The diabetes medication adherence clinic is handled by a pharmacist, focusing on improving medication adherence and glycaemic control through counseling and education.

### Sample size and sampling

The EnPHC-Eva study evaluated its outcomes for T2D using two approaches, i.e. interrupted time series (ITS) and difference-in-differences (DiD). The sample size was calculated separately for both approaches. In general, the minimum number of data points required for interrupted time-series analysis is 12 time points (six before and six after the intervention) with a minimum of 50 observations per time point [17]. In EnPHC-Eva study, we estimated a minimum 400 cases (10 cases per clinic) per time point for eight consecutive months before and after the intervention for practical and feasibility reasons. For the second approach, estimation of sample size for DiD was based on 28% effect size, 80% power, alpha value of 0.05 and cluster effect of 0.091. In total, the minimum baseline sample size required was 5200 T2D cases: 2000 for DiD and 3200 for ITS. We further adjusted the minimum required number to account for 40% potentially unavailable records. At the time of analysis, only data from the first 6 months were available and the data for the remaining 2 months would be collected during the next phase of data collection (post-intervention) between April and May 2018 due to logistic and time-constraint issues during the first phase of data collection. The cases were sampled each month by systematic random sampling of patient medical records and data was extracted into an electronic structured data collection form using mobile tablets.

### Patients

Patients aged 30 and above, diagnosed with T2D, had a clinic visit for T2D between 01 Nov 2016 and 30 April 2017 and had at least one HbA1c, SBP and LDL-C measurement within 1 year prior to the date of visit were included for analysis. Pregnant women with diabetes were excluded because disease management for gestational diabetes differs from non-pregnant patients.

### Variables

Outcome measures of this study were the most recent HbA1c, SBP and LDL-C values. The 2015 Malaysian Clinical Practice Guideline for T2D recommends the following treatment targets: HBA1c ≤ 7.0%, blood pressure ≤ 135/75 mmHg and LDL-C ≤ 2.6 mmol/L for most patients with T2D [18]. The following patient characteristics were included in the analysis based on literature as predictors of control of intermediate clinical outcomes in T2D [19–24]: patient age, sex, ethnicity, body mass index (BMI), duration of T2D, presence of hypertension and hyperlipidaemia, presence of T2D complications, insulin use, antihypertensive and statin (HMG-CoA reductase inhibitors). Complications of T2D were categorised by microvascular and macrovascular complications. Microvascular complications included nephropathy (proteinuria or chronic kidney disease), retinopathy, cataract, neuropathy (unspecified neuropathy, erectile dysfunction, foot ulcer or amputation) while macrovascular complications were coronary heart disease (myocardial infarction, angina, acute coronary syndrome and coronary artery stenosis), heart failure, cerebrovascular disease (stroke and transient ischaemic attack) and peripheral vascular disease. Glucose-lowering medications, number of antihypertensive as well as lipid-lowering medication were included in the final regression because of their effect on HbA1c control. Angiotensin-converting enzyme inhibitors (ACEI) were found to improve insulin sensitivity [23] while statins (HMG-CoA reductase inhibitors) were reported to be associated with increase in HbA1c [25].

To explain potential variation due to between clinic differences, the clinic level characteristics captured were geographical location (urban or rural), number of clinic attendances per day, availability of a full-time FMS in the clinic, availability of at least one full time diabetes educator in the clinic and availability of diabetes medication adherence services.

### Statistical analysis

Continuous variables were presented as mean and standard deviation while categorical variables were reported in frequencies and percentages. Statistical significance (alpha) was set at 0.05 for all comparisons.

Multilevel linear regression models were constructed for each outcome. When patients are grouped within clusters such as clinics, outcomes for those within the same cluster are more similar when compared to a patient from another clinic because of exposure to a common contextual effect [14]. Multilevel analysis accounts for the hierarchical structure of the data where patients (level 1) were nested within clinics (level 2) and is able to partition and quantify the amount of variation occurring at each level. Hence, we were able to identify the level where greatest variation lies for each outcome. Missing data rates ranged from 0.06 to 33%. Missing values were highest for the outcomes of interest, where 1150 (21%) and 1762 (33%) of patients did not have data for HbA1c and LDL-C values respectively. The data did not contain additional auxiliary variables which could be used to impute these missing outcomes through multiple imputation, hence we conducted complete case analysis for all models. We constructed the multilevel model by increasing complexity: first, we built an empty model with only a random intercept. Subsequently, we included the patient variables and the final model includes both patient and clinic variables.

For the regression analyses, we intended to interpret the intercept (or constant) for each of the models. The intercept gives the expected mean outcome values for HbA1c, SBP and LDL-C for the study sample when all predictors, X are equal to zero. For categorical variables, X = 0 refers to reference category for each variable. However, zero is not a meaningful value for continuous variables such as age and BMI. Therefore, we centred all eight continuous predictors in the models on their respective means, such that the value of 0 for these centred variables now refer to grand mean of the study sample [26].

Additionally, caterpillar plots were created to visualize the differences between adjusted clinic means for each outcome. Clinic estimates with 95% confidence intervals (95% CI) from the fully adjusted models were plotted. We calculated the intra-class correlation coefficient (ICC) to quantify the proportion of clinic variance of the total variance for all outcomes, where

14 $$ ICC=\frac{variance\ between\ clinics}{\left( variance\ between\ clinics+ variance\ within\ clinics\right)} $$

We used likelihood ratio tests to compare model fit between single-and multilevel models for each outcome. Improvement in goodness of fit is reflected in the reduction of ‘deviance’ statistics as variables were introduced consecutively into the models [27, 28]. The parameters of the multilevel regression were generated using maximum likelihood estimation. Visual inspection of residual plots was done and no obvious deviations from homoscedasticity or normality were observed. All variables were also checked for multicollinearity and no predictor pairs were found to be collinear (variance inflation factors range between 1.02 and 1.64). Data analyses were conducted using R version 3.6.1 [29]. The lme4 package was used for mixed effect modelling while the ggplot2 was used to generate the caterpillar plots [30, 31].

## Results

Out of 5425 patients with T2D we included 2960 patients who had complete data for all variables for the final regression model. Patient and clinic characteristics are presented in Table 1. The study population had a mean age of 60 years, was predominantly female (63.3%) and had a mean duration of T2D of 7.3 years. Seventy-nine percent of patients had hypertension while 52% had hyperlipidaemia. Micro- and macrovascular complications were present 28 and 8% of patients, respectively. On pharmacological management, 31.3% of patients were on insulin therapy, 66.3% were given either ACEI or ARBs for management of hypertension and about 81.1% of patients were on statins. There were also a percentage of patients who did not receive pharmacotherapy for glucose-, blood pressure- and lipid-lowering. Three percent of patients did not receive any glucose-lowering therapy and three quarter of these patients (75%) had HbA1c levels that were within target range (<=7%). As for the 12.8% of patients who did not receive any antihypertensive agent, about 13% of them had blood pressure above the national practice guideline target of 135/75 mmHg on two separate clinic visits [18]. On average, patients were obese with a mean BMI of 28.3 kg/m^2^ and had mean HbA1c of 8.4%, mean SBP of 137.7 mmHg and mean LDL-C of 3.0 mmol/L. The clinics in this study were largely located in urban areas (55%). A quarter of them had full-time family medicine specialists, 60% had permanent diabetes educators and 85% provided diabetes medication adherence services.

**Table 1:** Patient and clinic characteristics

Characteristics	N (%)
Demographics (*n* = 2960)	
Age (years) ^a^	60 (11)
Sex	
Male	1085 (36.7%)
Female	1875 (63.3%)
Ethnicity	
Malay	2098 (70.9)
Chinese	486 (16.4)
Indian	357 (12.1)
Others	19 (0.6)
Diabetes variables and comorbidities	
Duration of diabetes (years) ^a^	7.3 (5.6)
Hypertension	2341 (79.1)
Hyperlipidaemia	1552 (52.4)
Microvascular complications	830 (28.0)
Nephropathy	401 (13.5)
Diabetic eye complications (retinopathy, cataract)	303 (10.2)
Neuropathy	126 (4.3)
Macrovascular complications	236 (8.0)
Coronary heart disease	145 (4.9)
Cerebrovascular disease	57 (1.9)
Heart failure	33 (1.1)
Peripheral vascular disease	1 (0.03)
Glucose-lowering therapy	
Oral only	1942 (65.6)
Insulin ± oral	927 (31.3)
No pharmacotherapy	91 (3.1)
Antihypertensive therapy	
ACEI or ARB	1962 (66.3)
Other antihypertensive	618 (20.9)
No pharmacotherapy	380 (12.8)
Lipid-lowering therapy	
Statin ± other lipid-lowering agent	2401 (81.1)
Other lipid-lowering agent only	49 (1.7)
No pharmacotherapy	510 (17.2)
Clinical and laboratory measures	
Body mass index (BMI, kg/m^2^) ^a^	28.3 (5.9)
Systolic blood pressure (mmHg) ^a^	137.7 (18.7)
Diastolic blood pressure (mmHg) ^a^	77.7 (10.6)
HbA1c (%) ^a^	8.4 (2.2)
Total cholesterol (mmol/L) ^a^	5.1 (1.2)
Low-density lipoprotein cholesterol (mmol/L) ^a^	3.0 (1.1)
High-density lipoprotein cholesterol (mmol/L) ^a^	1.3 (0.6)
Clinic characteristics (*n* = 40)	
Geographical location	
Urban	22 (55.0)
Rural	18 (45.0)
Daily attendances ^a^	210 (160)
Family medicine specialist available	10 (25.0)
Diabetes educator available	24 (60.0)
Diabetes medication adherence service available	34 (85.0)

*ACEI* angiotensin-converting enzyme inhibitor, *ARB* angiotensin-II receptor blocker, *HbA1c* glycated haemoglobin;

^a^mean (standard deviation)

**Fig. 1:**
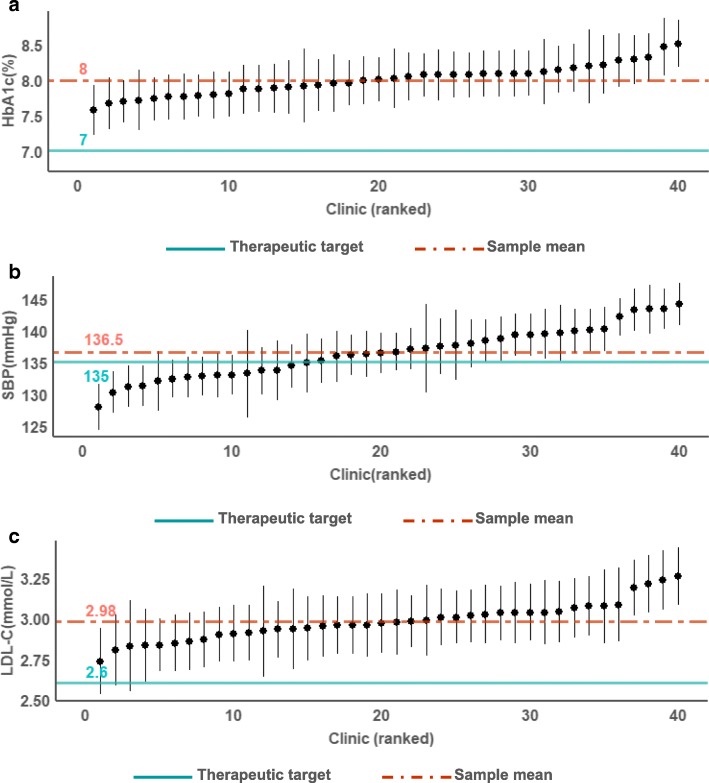
**a** Mean clinic HbA1c estimates with 95% CI after adjustment for patient and clinic characteristics. The dash-dotted line represents the mean of all clinics while the solid line represents the therapeutic target range recommended by the national clinical practice guideline. **b** Mean clinic SBP estimates with 95% CI after adjustment for patient and clinic characteristics. The dash-dotted line represents the mean of all clinics while the solid line represents the therapeutic target range recommended by the national clinical practice guideline. **c**. Mean clinic LDL-C estimates with 95% CI after adjustment for patient and clinic characteristics. The dash-dotted line represents the mean of all clinics while the solid line represents the therapeutic target range recommended by the national clinical practice guideline

The absolute and percentage variance attributable to patient and clinic levels were displayed for each outcome in Table 2. Results from the linear multilevel models show that variation in all three intermediate outcome measures occurs predominantly at the patient level, ranging between 93 and 98% (Table 2), after adjusting for patient and clinic characteristics. Conversely, between clinic differences accounts for a small but significant percentage of the total variance in HbA1c, SBP and LDL-C values. Figures 1a, b and c show the estimates and 95% CI by each clinic for HbA1c, SBP and LDL-C respectively. The adjusted mean levels for all outcomes were denoted by the dash-dotted red line where HbA1c is 8.0%, SBP is 136.5 mmHg and LDL-C is 2.98 mmol/L, were above targets recommended by the national clinical practice guideline, denoted by blue solid lines in Fig. 1 [18]. Among the three, HbA1c and LDL-C are almost equally furthest from therapeutic targets i.e. both measures are on average 14 and 15% above their recommended targets. Additionally, for both measures, there were few clinics which conclusively differed from the overall mean. In contrast, bigger differences between clinics was observed when it comes to SBP and this is reflected in the larger number of clinics which performed better and worse than average (Fig. 1b) and the higher ICC values compared to the other outcomes (ICC 0.07 vs 0.02) reported in Table 2.

**Table 2:** Absolute and percent of variance in HbA1c, SBP and LDL-C attributable to clinic and patient levels

Random effects	Fully adjusted model		
	HbA1c	SBP	LDL-C
Variance component	Variance (SD)	Variance (SD)	Variance (SD)
Clinic level	0.09 (0.30)	22.78 (4.77)	0.03 (0.16)
Patient level	3.63 (1.91)	285.63 (16.90)	1.05 (1.02)
Percent of total variance			
Clinic level	2.4%	7.4%	2.4%
Patient level	97.6%	92.6%	97.6%
Intracluster correlation coefficient (ICC)	0.02	0.07	0.02

Two-level models with a random effect for clinic. All models were adjusted for patient and clinic characteristics. [Likelihood ratio test results showed that two-level models produced better fit compared to single-level models for all three outcomes]

*HbA1c* glycated haemoglobin, *LDL-C* low-density lipoprotein cholesterol, *SBP* systolic blood pressure, *SD* standard deviation

Inclusion of patient characteristics into the empty model for HbA1c explained 14 and 26% of variance between clinics and between patients respectively (Additional file 1: Table S1). In contrast to the HbA1c model, addition of patient characteristics into the empty model for SBP explained slightly more variance between clinics (16%) but less of the variance among patients within clinics (15%) (Additional file 1: Table S2). Similarly, incorporating patient variables into the linear multilevel model for LDL-C explained more of the variance occurring at clinic level (34%) than those between patients (4%) (Additional file 1: Table S3). Overall, we found that for all three outcomes, the inclusion of clinic characteristics into the models had only marginally explained the variance at both between and within clinic levels (Additional file 1: Tables S1, S2, and S3).

Table 3 presents the coefficients, 95% CI and statistical significance for the linear multilevel models which included patient and clinic level characteristics. Increase in patient age is associated with lower levels in HbA1c and LDL-C but higher SBP. Proxy measures for disease severity such as duration of diabetes, microvascular complications, being on insulin and number of antihypertensives display the strongest association with poor control in HbA1c, SBP and LDL-C. Further, there is general correlation between all three intermediate clinical measures, where patients who are uncontrolled for one outcome is more likely to be uncontrolled for another intermediate outcome except for the relationship between SBP and HbA1c. Interestingly, none of the clinic level predictors including availability of a family medicine specialist and diabetes educator influenced control of all three outcome measures.

**Table 3:** Patient and clinic determinants of HbA1c, SBP and LDL-C levels in T2D

Fixed effects	HbA1c		SBP		LDL-C	
	Coefficient (95% CI)	*p*-value	Coefficient (95% CI)	*p*-value	Coefficient (95% CI)	*p*-value
Patient level						
Age (years)	− 0.04(− 0.05, − 0.03))	***	0.08(0.01, 0.15)	*	− 0.007(− 0.01, − 0.003)	**
Male sex	− 0.04(− 0.19, 0.10)		−1.92(−3.22, − 0.63)	**	− 0.05(− 0.12, 0.03)	
Malay ethnicity	0.01(− 0.15, 0.18)		1.85(0.35, 3.35)	*	0.27(0.18, 0.36)	***
Duration of diabetes (years)	0.05(0.04, 0.07)	***	− 0.09(− 0.21, 0.04)		− 0.01(− 0.02, − 0.003)	**
Body mass index	0.004(− 0.01, 0.02)		0.06(− 0.06, 0.17)		− 0.01(− 0.02, − 0.004)	**
HbA1c	–		−0.22(− 0.54, 0.11)		0.07(0.05, 0.09)	***
SBP	− 0.005(− 0.009, − 0.001)	**	–		0.002(0.0001, 0.004)	*
LDL-C	0.23(0.16, 0.29)	***	0.67(0.07, 1.26)	*	–	
Hypertension	–		2.64(0.85, 4.43)	**	–	
Hyperlipidaemia	–		–		0.04(−0.04, 0.13)	
Microvascular complication	0.21(0.03, 0.38)	*	1.79(0.19, 3.38)	*	−0.10(− 0.19, − 0.0001)	
Macrovascular complication	− 0.07(− 0.34, 0.20)		−1.23(−3.64, 1.18)		−0.14(− 0.28, 0.01)	
Insulin	1.81(1.65, 1.98)	***	0.25(−1.31, 1.81)		−0.01(− 0.11, 0.08)	
Number of antihypertensive(s)	–		4.43(3.76, 5.10)	***	0.03(−0.01, 0.07)	
ACEI/ARB	0.05(−0.10, 0.20)		–		–	
Statin	−0.06(− 0.24, 0.12)		−2.56(−4.17, − 0.29)	**	−0.01(− 0.11, 0.09)	
Clinic level						
Urban geographical location	−0.11(− 0.41, 0.19)		−3.03(−7.12, 1.05)		0.04(− 0.12, 0.19)	
Daily attendances	−0.0003(− 0.001, 0.001)		0.01(− 0.01, 0.02)		0.00003(− 0.0004, 0.0005)	
Family medicine specialist available	−0.05(− 0.40, 0.30)		− 0.41(−5.20, 4.39)		−0.04(− 0.23, 0.14)	
Diabetes educator available	−0.15(− 0.41, 0.12)		0.03(−3.68, 3.73)		− 0.04(− 0.18, 0.10)	
Diabetes medication adherence service available	− 0.04(− 0.40, 0.33)		2.04(−2.99, 7.07)		−0.13(− 0.33, 0.06)	
Goodness of fit						
Deviance^a^	12,242		25,193		8551	
Deviance change from empty model	− 900		− 384		− 148	

*p < 0.05, **p < 0.01, ***p < 0.001

*ACEI* angiotensin-converting enzyme inhibitor, *ARB* angiotensin-II receptor blocker, *CI* confidence interval, *HbA1c* glycated haemoglobin, *LDL-C* low-density lipoprotein cholesterol, *SBP* systolic blood pressure, *T2D* type 2 diabetes

^a^Decrease in ‘deviance’ reflects improvement in goodness of fit of the final model compared to the empty model. Change in deviance with each consecutive model were shown in the Additional file: Table

## Discussion

One of the aims to achieving better health care quality is to reduce unnecessary variation in disease management and outcomes. We found that greatest variation in intermediate clinical outcomes for T2D lie within clinics, at the patient level. This is consistent with findings by O′ Connor et al. and Charalampopoulos et al., where clinic level variation account for only a small percentage of the total variance in glycaemic control [10, 32]. There were relatively few clinics which performed worse than average for all three outcomes; hence focusing interventions on only those with poor performance will not be very efficient. Despite the small variability in treatment outcomes between clinics, intervening at the clinic and health provider level may still be useful and practical because these levels are more directly accessible than individual patients [33]. Moreover, there is still a clear gap between mean performances and national therapeutic targets for HbA1c and LDL-C control. These therapeutic targets of less than or equal 7% and 2.6 mmol/L for HbA1c and LDL-C are also consistent to those recommended by the International Diabetes Federation [34]. The results highlight an opportunity for closing this performance and target gap by improving disease management practices at the clinic level. Given the low variability in performance across clinics, our findings support the use of standard initiatives across all clinics to push disease control towards treatment targets.

Homogeneity in HbA1c, SBP and LDL-C levels observed between clinics can be explained by similarities in infrastructure and resources as they are managed under a single administration, the Ministry of Health. Although each clinic may have different delivery system designs [6], a lack of differences in treatment outcomes at the clinic level suggests that uniform interventions may be applied to all clinics to shift overall outcome to meet targets. The strategies that have been shown to improve intermediate patient outcomes include provider feedback, performance measurement, public reporting, financial incentives and benchmarking between clinics or individual providers [35, 36]. Much of the variability in HbA1c, SBP and LDL-C levels are attributable to the differences between patients. After adjusting for patient and clinic characteristics, most of the unexplained variation for HbA1c, SBP and LDL-C remain at the patient level. This is potentially due to other patient determinants such as medication adherence, socioeconomic status, health beliefs and patient self-care practice that were not captured in this study. Two things are implied from this finding. First, it is necessary for health providers to personalize therapeutic strategies based on individual patients. Second, patients need to be held accountable for their disease control. Patient-centered approaches include empowerment and engagement in treatment decision-making and self-care, use of reminder systems, self-monitoring of diabetes and promotion of diet, behavioural and lifestyle modifications [8]. Whilst we know that most differences in treatment outcomes reside within patients, it is the joint partnerships formed between patients and multi-disciplinary providers that are most likely to effect change [32].

Between the three outcomes evaluated, HbA1c and LDL-C control offers the largest potential for improvement from the current adjusted mean levels to clinical guideline recommended targets [18]. And yet this gap between actual performance and therapeutic targets is evident although 97 and 83% of patients are already on pharmacotherapy to lower glucose and lipid levels. These findings suggest the importance of other components of diabetes care such as treatment intensification, medication adherence, patient’s health beliefs, weight management, dietary intake and physical activity in improving disease control [6]. Further studies using the qualitative approaches may be conducted among health providers and patients to identify other barriers to disease control and develop targeted strategies to achieve better outcomes. Optimal disease management involves a complex interaction between providers and patients. Patient self-care and shared decision-making are recognized as a crucial part of diabetes care [36] and this task of empowering patients to take charge of their disease is complicated by low health literacy and the multicultural characteristics of patients in Malaysia [37, 38]. Thus, diabetes education needs to go beyond basic knowledge in diabetes and take into account cultural, psychosocial and family support aspects of individual patients [38, 39]. It is also known that people with diabetes in Malaysia consume diets high in carbohydrates and fat while more than half are physically inactive [6, 40]. These factors together with overweight or obesity contributed not only to the high prevalence of DM in the country but also poor disease control. In summary, health initiatives for T2D should be taken from two respect; one from improving the way health providers manage diabetes at the clinic level and another from community health perspective to address dietary and physical activity concerns.

We investigated the factors that could influence the outcomes by including patient and clinic characteristics in the multilevel models. Age, sex and ethnicity showed inconsistent effects for the three clinical outcomes. This finding is in agreement with a systematic review and a study by Frei et al. evaluating the impact of patient characteristics on diabetes outcome indicators [20, 41] where the authors found inconsistent impact for demographic characteristics. Despite known differences in prevalence of diabetes by ethnicity [6], it appears that disease control does not depend on these demographic characteristics but rather individual unmeasured factors related to individual health beliefs and lifestyles. The same systematic review mentioned above also did not show consistent influence of comorbidity and diabetes duration on HbA1c, SBP and LDL-C levels [20]. Contrastingly, we found that diabetes duration, presence of microvascular complications, being treated with insulin and number of antihypertensives were associated with poorer disease control. These predictors were likely a reflection of disease progression of diabetes in these patients. Further, we noted that poor control on one outcome predicts poor control of another intermediate outcome for diabetes, particularly the HbA1c and LDL-C pair. This observation is in line with a study by Jackson et al. which found modest association between LDL-C control and HbA1c control [42]. Our findings suggest a potential synergistic effect where control of one outcome increases the likelihood for control of the other and that simultaneous control of intermediate outcomes is more likely to be achieved when either one of the outcomes are within control.

None of the clinic level characteristics included in the model influenced HbA1c, SBP and LDL-C control. Kahn and colleagues demonstrated that having a certified diabetes educator within the primary care team resulted in improvement in Hba1c control [43]. It is interesting to note that neither having a diabetes educator nor medication adherence services in clinics influenced glycaemic outcomes. On the former, there are several possible reasons; (i) lack of standardised training modules for diabetes educators, (ii) lack of a pre-defined set of activities and key targets for the role of a diabetes educator, and (iii) multi-tasking, where the diabetes educator may also need to take on other roles in the provision of primary care services [6]. An approach would be to standardise the delivery of diabetes education, through accreditation programs for these services in the country. As for medication adherence service; its lack of impact on outcomes despite the availability of a standardised program [44] may be due to the small proportion of total diabetes patients which received the service. Based on information from the same data as the present study, only 8% of all T2D patients had ever received the medication adherence service [unpublished data from EnPHC-Eva]. This may be attributable to shortage of pharmacists to cater the service to a larger group of patients. More research is warranted to assess the quality of care provided by diabetes educators and pharmacists in the aspect of diabetes education and medication adherence services in primary care to identify areas for improvement. Whilst financial barrier is a known determinant for access to healthcare, it is unlikely to have an impact on this study’s results because treatment at public clinics comes at almost no cost to patients.

Few studies have quantified variation in intermediate clinical outcomes for T2D and a majority of these studies were done in high income countries [8, 32]. To our knowledge, this study is the first to evaluation clinic variation in diabetes outcomes in a middle-income nation. One of the strengths of this study is the use of multilevel models, which takes into account the hierarchical structure of the data and clustering within clinics. Further, data for this analysis was collected using an application with built-in validation rules to minimise data capture errors. There were several limitations in this study. First, we were unable to adjust for adherence to treatment because this information was not measured. About 45% of the patients had missing information on the outcome of interest and had to be omitted from the analysis. Therefore, we could not exclude the possibility of bias due to missing data. Also, there were 5 main categories of public health clinics Malaysia (categorised based on average daily patient attendances) but only 3 clinic types were involved in the implementation of the EnPHC interventions. The categories which were not represented in this study were the smallest and largest clinic types and this may partially explain the lack of variation found between clinics. We were also unable to disentangle provider level variation or control for provider characteristics as patients were not assigned to one single provider for all episodes of care but were managed by any provider who is on duty on the visit day. Also, it is possible that number of clinics may not be sufficiently powered to allow detection of effects for clinic characteristics [45].

## Conclusion

Clinic level variation in HbA1c, SBP and LDL-C accounts for a small percentage from total variation. More than 93% of variation in intermediate clinical outcomes in T2D is due to differences between patients. Among the three measures evaluated, HbA1c and LDL-C offers the largest room for improvement. Interventions need to be applied across all clinics, with a focus on customizing therapy based on individual patient characteristics. The predictors for poor control of intermediate diabetes outcomes are measures of disease progression including duration of diabetes, microvascular complications, being on insulin and number of antihypertensives. There is also small but significant association between the outcomes which suggests that simultaneous control is more likely to be achieved when one of the outcomes are within therapeutic targets.

## Supplementary information

**Additional file 1.** Detailed multilevel model results for HbA1c, SBP and LDL-C. The table includes the results for multilevel linear regression for the empty model, model with addition of patient variables and final model with patient and clinic variables. Both fixed and random effects are reported. Proportional changes in variance with the addition of variables and goodness of fit results are also included.

## Data Availability

Data for the current study was based on baseline information from the EnPHC evaluation study. Relevant aggregate data are presented within this paper and its supplementary information file. Due to ethical and confidentiality restrictions, individual data cannot be made publicly available. All requests for data access should be addressed to the Institute for Clinical Research at contact@crc.gov.my.

## Abbreviations

ACEI: Angiotensin-converting enzyme inhibitor
ARB: Angiotensin-II receptor blocker
BMI: Body mass index
CI: Confidence interval
DiD: Difference-in-differences
EnPHC: Enhanced Primary Healthcare Intervention Package
EnPHC-Eva: Enhanced Primary Healthcare Intervention Package Evaluation Study
FMS: Family medicine specialist
Hba1c: Glycated haemoglobin
ICC: Intracluster correlation coefficient
LDL-C: Low-density lipoprotein cholesterol
LMIC: Low- and middle-income countries
SBP: Systolic blood pressure
SD: Standard deviation
Statin: HMG-CoA reductase inhibitors
T2D: Type 2 diabetes
