# Supplementary material for: Clinic and patient variation in intermediate clinical outcomes for type 2 diabetes: a multilevel analysis
Source: BMC Fam Pract. 2019 Nov 15;20:158. doi: 10.1186/s12875-019-1045-1 (PMC6857311; doi:10.1186/s12875-019-1045-1)
Supplement: Supplementary file 1 — Additional file 1. Detailed multilevel model results for HbA1c, SBP and LDL-C. The table includes the results for multilevel linear regression for the empty model, model with addition of patient variables and final model with patient and clinic variables. Both fixed and random effects are reported. Proportional changes in variance with the addition of variables and goodness of fit results are also included. [file 12875_2019_1045_MOESM1_ESM.docx]

**Table S1. Detailed multilevel model results for HbA1c**

|  | **empty model** | **Model with patient variables** | **Model with patient and clinic variables** |
| --- | --- | --- | --- |
| **Fixed effects** | Coefficients (95%CI) | Coefficients (95%CI) | Coefficients (95%CI) |
| intercept | 8.42 (8.29,8.55) | 7.81 (7.56, 8.06) | 8.00 (7.53, 8.48) |
| **Patient level** |  |  |  |
| Age (years) |  | -0.04 (-0.05, -0.03) | -0.04 (-0.05, -0.03) |
| Male sex |  | -0.05 (-0.19, 0.10) | -0.04 (-0.19, 0.10) |
| Malay ethnicity |  | 0.003 (-0.16, 0.17) | 0.01 (-0.15, 0.18) |
| Duration of diabetes (years) |  | 0.05 (0.04, 0.07) | 0.05 (0.04, 0.07) |
| Body mass index (kg/m^2^) |  | 0.004 (-0.01, 0.02) | 0.004 (-0.01, 0.02) |
| HbA1c |  | - | - |
| SBP |  | -0.01 (-0.01, -0.001) | -0.005 (-0.009, -0.001) |
| LDL-C |  | 0.23 (0.16, 0.29) | 0.23 (0.16, 0.29) |
| Microvascular complication |  | 0.20 (0.03, 0.38) | 0.21 (0.03, 0.38) |
| Macrovascular complication |  | -0.08 (-0.35, 0.19) | -0.07 (-0.34, 0.20) |
| Insulin |  | 1.81 (1.65, 1.98) | 1.81 (1.65, 1.98) |
| Number of antihypertensive(s) |  | - | - |
| ACEI/ARB |  | 0.05 (-0.10, 0.20) | 0.05 (-0.10, 0.20) |
| Statin |  | -0.05 (-0.23, 0.13) | -0.06 (-0.24, 0.12) |
| **Clinic level** |  |  |  |
| Urban geographical location |  |  | -0.11 (-0.41, 0.19) |
| Daily attendances |  |  | -0.0003 (-0.001, 0.001) |
| Family medicine specialist available |  |  | -0.05 (-0.40, 0.30) |
| Diabetes educator available |  |  | -0.15 (-0.41, 0.12) |
| Diabetes medication adherence service available |  |  | -0.04 (-0.40, 0.33) |
| **Random effects** | **Variance (SD)** | **Variance (SD)** | **Variance (SD)** |
| Clinic | 0.10 (0.32) | 0.09 (0.30) | 0.09 (0.30) |
| Patient | 4.90 (2.21) | 3.63 (1.91) | 3.63 (1.91) |
| Intracluster correlation coefficient (ICC) | 0.02 | 0.02 | 0.02 |
| **Proportional change in variance** |  |  |  |
| Clinic | - | -14.47% | 2.31% |
| Patient | - | -25.88% | -0.01% |
| **Goodness of fit** |  |  |  |
| Deviance | 13142 | 12247 | 12239 |
| Deviance change from empty model^a^ | - | -895 | -903 |

1. Deviance change from empty model to model with patient variables is statistically significant (ꭓ2(12) = 894.9, p<0.001) while deviance change from model with patient variables to model with patient and clinic variables was not statistically significant (ꭓ2(5) = 5.4, p=0.4).

**Table S2. Detailed multilevel model results for systolic blood pressure (SBP)**

|  | **empty model** | **Model with patient variables** | **Model with patient and clinic variables** |
| --- | --- | --- | --- |
| **Fixed effects** | Coefficients (95%CI) | Coefficients (95%CI) | Coefficients (95%CI) |
| intercept | 137.50 (135.74, 139.26) | 136.52 (133.74, 139.30) | 136.50 (130.06, 142.94) |
| **Patient level** |  |  |  |
| Age (years) |  | 0.08 (0.01, 0.15) | 0.08 (0.01, 0.15) |
| Male sex |  | -1.92 (-3.21, -0.63) | -1.92 (-3.22, -0.63) |
| Malay ethnicity |  | 1.96 (0.42, 3.41) | 1.85 (0.35, 3.35) |
| Duration of diabetes (years) |  | -0.09 (-0.21, 0.04) | -0.09 (-0.21, 0.04) |
| Body mass index (kg/m^2^) |  | 0.06 (-0.06, 0.17) | 0.06 (-0.06, 0.17) |
| HbA1c |  | -0.22 (-0.54, 0.11) | -0.22 (-0.54, 0.11) |
| SBP |  | - | - |
| LDL-C |  | 0.66 (0.06, 1.26) | 0.67 (0.07, 1.26) |
| Hypertension |  | 2.59 (0.81, 4.38) | 2.64 (0.85, 4.43) |
| Microvascular complication |  | 1.78 (0.19, 3.37) | 1.79 (0.19, 3.38) |
| Macrovascular complication |  | -1.24 (-3.65, 1.17) | -1.23 (-3.64, 1.18) |
| Insulin |  | 0.25 (-1.31, 1.81) | 0.25 (-1.31, 1.81) |
| Number of antihypertensive(s) |  | 4.43 (3.76-5.10) | 4.43 (3.76, 5.10) |
| ACEI/ARB |  | - | - |
| Statin |  | -2.59 (-4.20, -0.98) | -2.56 (-4.17, -0.95) |
| **Clinic level** |  |  |  |
| Urban geographical location |  |  | -3.03 (-7.12, 1.05) |
| Daily attendances |  |  | 0.01 (-0.01, 0.02) |
| Family medicine specialist available |  |  | -0.41 (-5.20, 4.39) |
| Diabetes educator available |  |  | 0.03 (-3.68, 3.73) |
| Diabetes medication adherence service available |  |  | 2.04 (-2.99, 7.07) |
| **Random effects** | **Variance (SD)** | **Variance (SD)** | **Variance (SD)** |
| Clinic | 26.83 (5.29) | 22.42 (4.74) | 22.78 (4.77) |
| Patient | 337.9 (18.38) | 285.64 (16.90) | 285.63 (16.90) |
| Intracluster correlation coefficient (ICC) | 0.07 | 0.07 | 0.07 |
| **Proportional change in variance** |  |  |  |
| Clinic | - | -16.44% | 1.61% |
| Patient | - | -15.47% | 0% |
| **Goodness of fit** |  |  |  |
| Deviance | 25577 | 25198 | 25193 |
| Deviance change from empty model^a^ | - | -379 | -384 |

1. Deviance change from empty model to model with patient variables is statistically significant (ꭓ2(13) = 378.33, p<0.001) while deviance change from model with patient variables to model with patient and clinic variables was not statistically significant (ꭓ2(5) = 5.1, p=0.4).

**Table S3. Detailed multilevel model results for low-density lipoprotein cholesterol (LDL-C)**

|  | **empty model** | **Model with patient variables** | **Model with patient and clinic variables** |
| --- | --- | --- | --- |
| **Fixed effects** | Coefficients (95%CI) | Coefficients (95%CI) | Coefficients (95%CI) |
| intercept | 3.01 (2.95, 3.07) | 2.86 (2.73, 2.98) | 2.98 (2.77, 3.24) |
| **Patient level** |  |  |  |
| Age (years) |  | -0.01 (-0.01, -0.003) | -0.007 (-0.01, -0.003) |
| Male sex |  | -0.05 (-0.12, 0.03) | -0.05 (-0.12, 0.03) |
| Malay ethnicity |  | 0.27 (0.18, 0.36) | 0.27 (0.18, 0.36) |
| Duration of diabetes (years) |  | -0.01 (-0.02, -0.003) | -0.01 (-0.02, -0.003) |
| Body mass index (kg/m^2^) |  | -0.01 (-0.02, -0.004) | -0.01 (-0.02, -0.004) |
| HbA1c |  | 0.07 (0.05, 0.09) | 0.07 (0.05, 0.09) |
| SBP |  | 0.002 (0.00004, 0.004) | 0.002 (-0.0002, 0.004) |
| LDL-C |  | - | - |
| Hyperlipidaemia |  | 0.04 (-0.04, 0.13) | 0.04 (-0.04, 0.13) |
| Microvascular complication |  | -0.10 (-0.19, -0.004) | -0.10 (-0.19, -0.0001) |
| Macrovascular complication |  | -0.14 (-0.28, 0.01) | -0.14 (-0.28, 0.01) |
| Insulin |  | -0.02 (-0.11, 0.07) | -0.01 (-0.11, 0.08) |
| Number of antihypertensive(s) |  | 0.04 (-0.002, 0.08) | 0.03 (-0.01, 0.07) |
| ACEI/ARB |  | - |  |
| Statin |  | -0.01 (-0.11, 0.09) | -0.01 (-0.11, 0.09) |
| **Clinic level** |  |  |  |
| Urban geographical location |  |  | 0.04 (-0.12, 0.19) |
| Daily attendances |  |  | 0.00003 (-0.0004, 0.0005) |
| Family medicine specialist available |  |  | -0.04 (-0.23, 0.14) |
| Diabetes educator available |  |  | -0.04 (-0.18, 0.10) |
| Diabetes medication adherence service available |  |  | -0.13 (-0.33, 0.06) |
| **Random effects** | **Variance (SD)** | **Variance (SD)** | **Variance (SD)** |
| Clinic | 0.04 (0.19) | 0.02 (0.15) | 0.03 (0.16) |
| Patient | 1.09 (1.04) | 1.05 (1.02) | 1.04 (1.02) |
| Intracluster correlation coefficient (ICC) | 0.03 | 0.02 | 0.02 |
| **Proportional change in variance** |  |  |  |
| Clinic | - | -34.13 % | 6.70% |
| Patient | - | -4.02% | 4.53% |
| **Goodness of fit** |  |  |  |
| Deviance | 8699 | 8555 | 8551 |
| Deviance change from empty model^a^ | - | -144 | -148 |

1. Deviance change from empty model to model with patient variables is statistically significant (ꭓ2(13) = 144.2, p<0.001) while deviance change from model with patient variables to model with patient and clinic variables was not statistically significant (ꭓ2(5) = 3.5, p=0.6).
